# Origin, evolution, dispersal and global population genetic structure of *Carlavirus sigmasolani*

**DOI:** 10.3389/fpls.2025.1667771

**Published:** 2025-09-24

**Authors:** Jianlin Lei, Beibei Liang, Hongwei Yang, Bo Zhang

**Affiliations:** School of Agriculture and Bioengineering, Longdong University, Qingyang, Gansu, China

**Keywords:** Potato virus S, evolutionary history, global dissemination, Bayesian phylogeographic analyses, intercontinental transmission, population structure

## Abstract

*Carlavirus sigmasolani* (Potato virus S, PVS) is a globally distributed plant virus infecting cultivated potato (*Solanum tuberosum*), causing yield losses and reduced tuber quality in the host crop, yet its evolutionary history, global dissemination and population genetic structure remain incompletely understood. In this study, we conducted comprehensive phylogenetic and Bayesian phylogeographic analyses of PVS using all available complete genome and coat protein (CP) gene sequences from 35 countries. Genome-based phylogenetic reconstruction identified four major phylogroups (I–IV), with Phylogroup I comprising only Colombian isolates and Phylogroup IV showing the broadest geographic distribution. In contrast, CP gene-based analyses revealed seven phylogroups (I–VII), including regionally restricted Phylogroups V (Colombia) and VI (Ecuador), and the globally dominant Phylogroup VII. A time-scaled Bayesian phylogenetic framework estimated a mean substitution rate of 3.11 × 10^-4^ substitutions/site/year (95% HPD: 2.19 × 10^-4^–4.07 × 10^-4^), and dated the most recent common ancestor (tMRCA) of PVS to approximately 1296 (95% HPD: 964–1578). Phylogeographic analysis based on CP gene sequences suggests that Ecuador is a likely center of origin for PVS, with intercontinental dissemination beginning in the 16th century and markedly accelerating during the 19th and 20th centuries. Iran and China were identified as major secondary hubs during this period, while Europe and the United States also contributed to global dissemination as important intercontinental transmission centers during the 20th and 21st centuries. Population genetic analyses indicated that South America retains the highest diversity, reinforcing its status as the center of origin, while the markedly lower diversity in Africa and Oceania suggests more recent introductions coupled with restricted gene flow. These data improve our understanding of PVS evolution, spread and population structure, supporting the development of effective monitoring and control strategies.

## 1 Introduction

Potato (*Solanum tuberosum*) ranks as the fourth most important food crop globally, following wheat, rice, and maize, and plays a vital role in global food security by providing a rich source of carbohydrates, essential nutrients, and dietary energy to populations worldwide (Ren et al., 2025). Nevertheless, potato production faces significant constraints from a diverse array of diseases, particularly those induced by viruses (Kreuze et al., 2020). More than 50 different viruses have been reported to infect potato crops, with *Carlavirus sigmasolani* (Potato virus S, PVS; family *Betaflexiviridae*, genus *Carlavirus*) recognized as one of the most prevalent and widely distributed (Kumar et al., 2022; Santillan et al., 2018). PVS has been documented in all major potato-producing countries, including China, India, Russia, the United States, Ukraine, and several countries in Europe, South America, and Africa (Wang et al., 2011; Halabi et al., 2021; Maslennikova et al., 2024; Halterman et al., 2012; Dmitruk et al., 2018; Duan et al., 2018; Santillan et al., 2018; Onditi et al., 2021), highlighting its significance as a persistent threat to potato production systems. Although PVS infections are often mild or latent, they are of particular concern due to their high prevalence and potential to remain undetected (Kreuze et al., 2020; Kumar et al., 2022; Brattey et al., 2002). When symptoms do manifest, they typically include mild mosaic patterns, slight leaf crinkling, and occasional interveinal chlorosis, with infected plants sometimes exhibiting reduced vigor and smaller tubers, especially in the presence of more virulent strains (Salazar, 1996; Wu et al., 2024; Li et al., 2018). While PVS alone generally causes limited yield losses, with reductions of up to 20% reported in severe cases, it frequently co-infects with other more common potato viruses such as *Potyvirus yituberosi* (Potato virus Y, PVY), *Potexvirus xylopotati* (Potato virus X, PVX), and *Polerovirus solani* (Potato leafroll virus, PLRV). Such co-infections can enhance symptom severity and lead to significantly greater reductions in tuber yield and quality (Nyalugwe et al., 2012; Topkaya et al., 2023). Moreover, the latent nature of many PVS infections poses a particular risk to seed potato certification systems, as asymptomatic plants may serve as undetected reservoirs for virus transmission (Kumar et al., 2020). PVS is primarily spread through infected seed tubers, enabling its persistence in vegetatively propagated crops (Halterman et al., 2012; Khurana, 2004). It can also be transmitted mechanically via farming tools and handling, and by aphids such as *Myzus persicae* and *Aphis gossypii* in a non-persistent manner, albeit with low efficiency. Additionally, grafting and transmission through botanical (true) seeds from infected maternal plants have also been reported. These multiple transmission pathways contribute to the widespread occurrence and long-term persistence of PVS in potato production systems (Wardrop et al., 1989; Bragard et al., 2020).

The genome of PVS is a single-stranded, positive-sense RNA of approximately 8.5 kb, encapsidated within flexuous filamentous particles. It contains six open reading frames (ORFs), flanked by 5′ and 3′ untranslated regions essential for replication and translation (Gutiérrez et al., 2013; Topkaya et al., 2023). ORF1 encodes a large replicase protein with conserved methyltransferase, helicase, and RNA-dependent RNA polymerase (RdRp) domains that drive viral genome replication. ORFs 2–4 constitute the triple gene block (TGB), encoding TGB1, TGB2, and TGB3, which cooperatively mediate cell-to-cell movement (Martelli et al., 2007). TGB1 also has RNA-binding activity and may suppress RNA silencing (Atabekova et al., 2023). ORF5 encodes the coat protein (CP), which is integral to virion assembly, systemic movement, and is commonly used as a diagnostic and phylogenetic marker, while ORF6 encodes a cysteine-rich nucleic acid-binding protein (CRP) implicated in pathogenicity and RNA silencing suppression during host-virus interactions (Topkaya et al., 2023; Bendahmane et al., 1995; Kumar et al., 2019; Lawrence and Jackson, 2001). Based on their ability to cause systemic infection in *Chenopodium* spp., PVS isolates from potato are biologically classified into two distinct strains: PVS^A^ (Andean) and PVS° (Ordinary). PVS^A^ is capable of inducing systemic infection in *Chenopodium* spp., while PVS° is restricted to causing only local lesions, without systemic movement within the host. The designation “PVS^A^” reflects the initial detection of these isolates in the Andean region of South America, which is also recognized as the center of origin for cultivated potato (Cox and Jones, 2010). Furthermore, PVS can be transmitted by aphids in a non-persistent manner, with the PVS^A^ reported to induce more pronounced foliar symptoms on potato plants compared to the PVS°, suggesting potential differences in pathogenicity and host interaction dynamics between the two strains (Slack, 1983).

With the advancement of sequencing technologies and the increasing availability of PVS gene sequences in GenBank from various countries, phylogenetic analyses have revealed that the traditional biologically defined strain names PVS° and PVS^A^ are no longer suitable for delineating evolutionary lineages (Topkaya et al., 2023). Such inconsistency arises from the finding that PVS^A^ isolates often cluster within the major PVS° phylogenetic clade, and conversely, PVS° isolates also appear within the PVS^A^ clade (Matoušek et al., 2005). To address this incongruity, the term PVS^CS^ (CS = *Chenopodium* systemic) was proposed to distinguish biologically defined PVS^A^ isolates from other genetically distinct members within the major PVS° phylogroup. Subsequently, the terms PVS^O-CS^ (CS = *Chenopodium* systemic) and PVS^A-CL^ (CL = *Chenopodium* localized) were proposed to designate isolates within the major PVS° phylogroup that are capable of systemic infection in *Chenopodium*, and those within the major PVS^A^ phylogroup that remain restricted to localized infection in inoculated leaves, respectively (Cox and Jones, 2010). To minimize confusion between biological and phylogenetic classifications, some researchers adopted a geography-based nomenclature. Sequencing of *Solanum phureja* isolates from Colombia revealed a distinct phylogenetic lineage, designated PVS^RVC^ (Duan et al., 2018). This lineage was later expanded to include additional Colombian and Ecuadorian isolates, and was termed the “second South American lineage” by Santillan et al (Santillan et al., 2018). In contrast, ten isolates collected from five countries within the Andean center of potato domestication (Bolivia, Chile, Colombia, Ecuador, and Peru) clustered within the previously defined PVS^A^ phylogroup, which was designated the “first South American lineage” (Santillan et al., 2018).

Although previous approaches helped distinguish PVS strains based on biological traits and phylogenetic relationships, the rapid accumulation of sequence data in GenBank has made classification increasingly challenging. In an effort to resolve this issue, a standardized Latin numeral system was proposed, in which PVS^I^, PVS^II^, and PVS^III^ correspond to the PVS°, PVS^A^, and PVS^RVC^ phylogroups, respectively, while retaining PVS° and PVS^A^ for biologically defined strains (Jones and Kehoe, 2016). Recently, Topkaya et al. applied this nomenclature system in a global phylogenetic analysis of PVS isolates; however, the expanding GenBank dataset has since outgrown this framework, limiting its suitability to reflect the current genetic diversity of the virus (Topkaya et al., 2023). Without addressing this nomenclature confusion, which is uncoupled from the underlying biology and genetics of PVS, comparisons across studies may become inconsistent and misleading. Here, we analyzed all available PVS complete genome and CP sequences to reconstruct molecular phylogenies and proposed an updated Latin numeral-based phylogroup classification. In addition, we further performed Bayesian evolutionary, population structure, and phylogeographic analyses to elucidate the global diversification and transmission patterns of PVS, thereby providing deeper insights into its evolutionary dynamics and informing the development of effective control strategies.

## 2 Material and methods

### 2.1 Genome sequence data collection

As of June 4, 2025, all available complete genome sequences (n = 152) (**Supplementary Table S1**) and CP gene sequences (n = 216; > 800 bp) of PVS were retrieved from the GenBank database (https://www.ncbi.nlm.nih.gov/genbank/). After data integration, a total of 368 CP gene sequences were obtained for subsequent analyses (**Supplementary Table S2**). Supplementary tables include information on the accession number, sampling date, isolate name, country of origin, and corresponding region for each isolate.

### 2.2 Phylogenetic tree construction

The complete genome and CP gene sequences of PVS were aligned using MAFFT v7.520. Subsequent manual refinement and trimming of the alignments were performed using MEGA7. The maximum likelihood (ML) tree was constructed using IQ-TREE v2.3.6 with 1,000 bootstrap replicates to assess branch support. The best-fit substitution models for the ML phylogenetic reconstruction were determined using ModelFinder implemented in IQ-TREE v2.3.6. For the complete genome of PVS, the optimal model was GTR+F+I+R3, while TVMe+I+G4 was selected as the best-fit model for the CP gene. The resulting phylogenetic trees were visualized and refined using FigTree v1.4.4.

### 2.3 Recombination detection

Potential recombination events in the complete genome and CP gene sequences of PSV were analyzed using the Recombination Detection Program (RDP) v4.101. The analysis was performed under default settings, employing multiple detection methods including RDP, GENECONV, BootScan, MaxChi, Chimaera, SiScan, and 3Seq. A sequence was considered recombinant only if recombination signals were identified by at least two of these methods.

### 2.4 Temporal signal evaluation

Temporal signal assessment of the PVS CP gene dataset was performed using root-to-tip regression analysis in TempEst v1.5.3, with sampling dates incorporated as temporal covariates (Rambaut et al., 2016). The initial analysis yielded a low coefficient of determination (R² = 0.0216), indicating a weak correlation between genetic divergence and sampling time. Despite this modest signal, the dataset was deemed suitable for Bayesian analyses under a relaxed molecular clock model. To enhance the temporal signal, we applied a filtering criterion by excluding sequences with an absolute residual greater than 0.015 based on the root-to-tip regression residuals. This led to the removal of five outlier sequences (MF496659, JX419379, KR152654, MK116550 and KY451037). In addition, recombination screening using RDP v4.101 identified one sequence (GU256061) as a potential recombinant. This sequence was also excluded to minimize confounding effects on molecular clock calibration. Following the removal of outliers and the recombinant sequence, a refined dataset comprising 362 CP gene sequences was obtained. Re-evaluation of the temporal signal in this filtered dataset yielded an improved R² value of 0.0771, supporting its application in subsequent Bayesian time-scaled phylogenetic analyses.

### 2.5 Bayesian time-scaled phylogenetic reconstruction

Time-scaled phylogenetic reconstruction was conducted using BEAST v10.5.0 with computational acceleration provided by the BEAGLE library (Drummond et al., 2002), employing an uncorrelated lognormal relaxed molecular clock model. The optimal nucleotide substitution model for Bayesian evolutionary analysis was determined using ModelFinder integrated in PhyloSuite v1.2.1. Based on Bayesian Information Criterion (BIC) scores, the generalized time-reversible model with empirical base frequencies and gamma-distributed rate heterogeneity (GTR+F+G4) was identified as the best-fitting model and subsequently applied in all BEAST analyses. Bayesian phylogenetic inference was performed in BEAST v10.5.0 under an Exponential Growth coalescent prior, selected as the most appropriate demographic model for the PVS CP gene dataset. Four independent Markov chain Monte Carlo (MCMC) runs of 4 × 10^8^ generations were executed, with parameters and trees sampled every 1,000 steps. Convergence and mixing were assessed using Tracer v1.7.2, ensuring effective sample sizes (ESS) greater than 200 for all estimated parameters. After discarding the initial 10% of samples as burn-in to remove pre-convergence states, the post-burn-in trees were combined, and a maximum clade credibility (MCC) tree was generated using TreeAnnotator v10.5.0. The resulting time-scaled MCC tree was annotated and visualized in FigTree v1.4.4, with node bars representing 95% highest posterior density (HPD) intervals for divergence time estimates. Within this Bayesian coalescent framework, BEAST was used to jointly estimate the evolutionary rate (substitution/site/year, s/s/y) and the time to the most recent common ancestor (tMRCA) for the PVS CP gene dataset.

### 2.6 Phylogeographic analysis

To infer the global transmission routes of PVS, a discrete trait phylogeographic analysis was conducted in BEAST v1.10.5 using an asymmetric transition model to characterize the spatial dynamics among geographic regions. The Bayesian MCMC analysis was run for 4 × 10^8^ generations, with parameters and trees sampled every 4,000 steps. The first 10% of samples were discarded as burn-in. Convergence and sampling efficiency were evaluated using Tracer v1.7.2, ensuring that all estimated parameters had ESS greater than 200. A Bayesian Stochastic Search Variable Selection (BSSVS) procedure was employed to identify statistically supported migration pathways. Bayes factors (BF) were calculated from the resulting log files to quantify support for individual diffusion routes, with BF values greater than 3 considered evidence of significant spatial linkage. The inferred migration routes were visualized using the spread.gl software (Li et al., 2024).

### 2.7 Population structure analysis

To understand the population genetic diversity of PVS across different geographical regions (Europe, Asia, Africa, North America, South America, and Oceania), we analyzed the CP gene sequences using DnaSP v.6.12.03 (Rozas et al., 2017). A series of molecular diversity parameters were estimated, including the number of haplotypes (h), haplotype diversity (Hd), the number of polymorphic (segregating) sites (S), the total number of mutations (η), the average number of nucleotide differences (k), and nucleotide diversity (π). In addition, genetic differentiation and gene flow among these regional populations were evaluated using the genetic differentiation index (Fixation index, Fst) and the number of migrants per generation (Nm).

## 3 Results

### 3.1 Phylogenetic analysis

**Figure 1:**
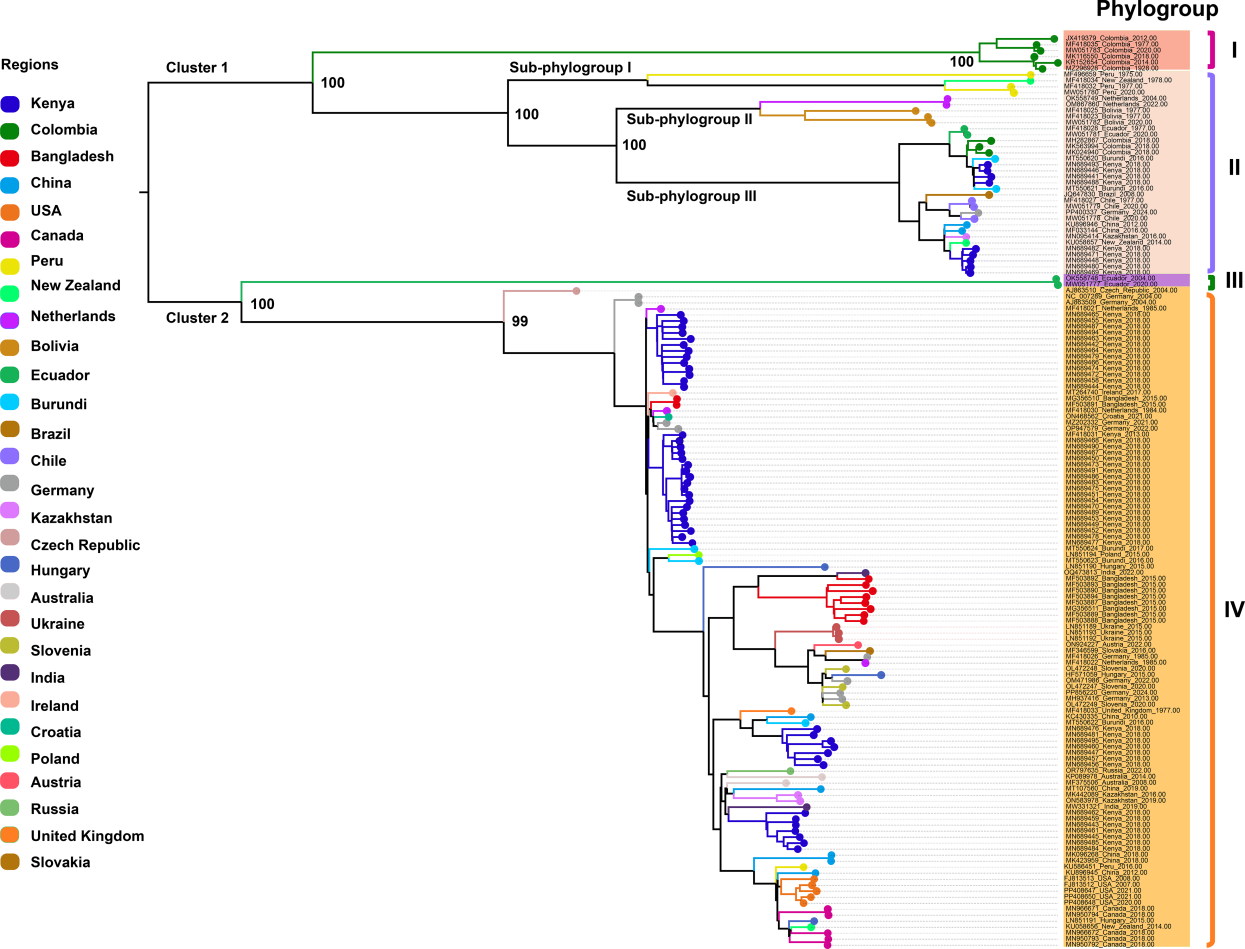
Maximum likelihood (ML) phylogenetic tree inferred from 152 complete genome sequences of PVS. An ML phylogenetic tree was constructed using IQ-TREE v2.3.6 under the GTR+F+I+R3 substitution model, with branch support assessed via 1,000 bootstrap replicates. Colors represent the geographic origin of the virus isolates.

We retrieved all available complete genome sequences (n = 152) of the virus from the GenBank database, representing isolates from 29 different countries worldwide. A ML phylogenetic tree was constructed based on these sequences (**Figure 1**). The resulting tree topology revealed two major clusters, designated as Cluster 1 and Cluster 2. Each cluster was further subdivided into two distinct phylogroups, referred to as Phylogroups I, II, III, and IV, respectively. Notably, Phylogroup I consisted exclusively of six isolates from Colombia, forming a genetically distinct Colombia-specific lineage. Phylogroup II was further divided into three well-supported sub-branches, designated as Sub-phylogroups I, II, and III. Sub-phylogroup I comprised four isolates originating from Peru and New Zealand. Sub-phylogroup II included five isolates, with two from the Netherlands and three from Bolivia. Sub-phylogroup III encompassed a more diverse set of 25 isolates collected from Ecuador, Colombia, Burundi, Kenya, Brazil, Chile, Germany, China, Kazakhstan, and New Zealand, indicating a broader geographic distribution and higher genetic diversity within this sub-lineage. Phylogroup III comprises two isolates from Ecuador, forming a distinct monophyletic lineage. Phylogroup IV consists of 110 isolates originating from 24 countries across multiple continents, including Africa (Kenya, Burundi), Asia (Bangladesh, China, Kazakhstan, India), Europe (Germany, Netherlands, Hungary, Ukraine, Slovenia, Czech Republic, Ireland, Croatia, Poland, Austria, Slovakia, United Kingdom, Russia), North America (USA, Canada), South America (Peru), and Oceania (Australia, New Zealand). Additionally, within Phylogroup IV, several country-specific monophyletic clades were also observed, suggesting localized diversification events and potential region-specific evolutionary trajectories. These data highlight the global diversity of PVS, with Phylogroup IV emerging as the most prevalent and widespread lineage, representing the dominant strain currently circulating worldwide.

To assess whether the CP gene can serve as a reliable proxy for the phylogenetic relationships inferred from complete genome sequences of PVS, we extracted CP gene sequences from the available complete genomes (n = 152) and constructed a ML phylogenetic tree (**Supplementary Figure S1**). The resulting CP-based tree topology differed notably from that of the full-genome phylogeny, revealing five distinct phylogroups, designated Phylogroups I–V (**Supplementary Figure S1**). Moreover, several isolates displayed incongruent phylogenetic placements relative to their positions in the whole-genome phylogeny. These discrepancies indicate that CP gene phylogeny alone does not fully capture the evolutionary history derived from complete genome analyses.

**Figure 2:**
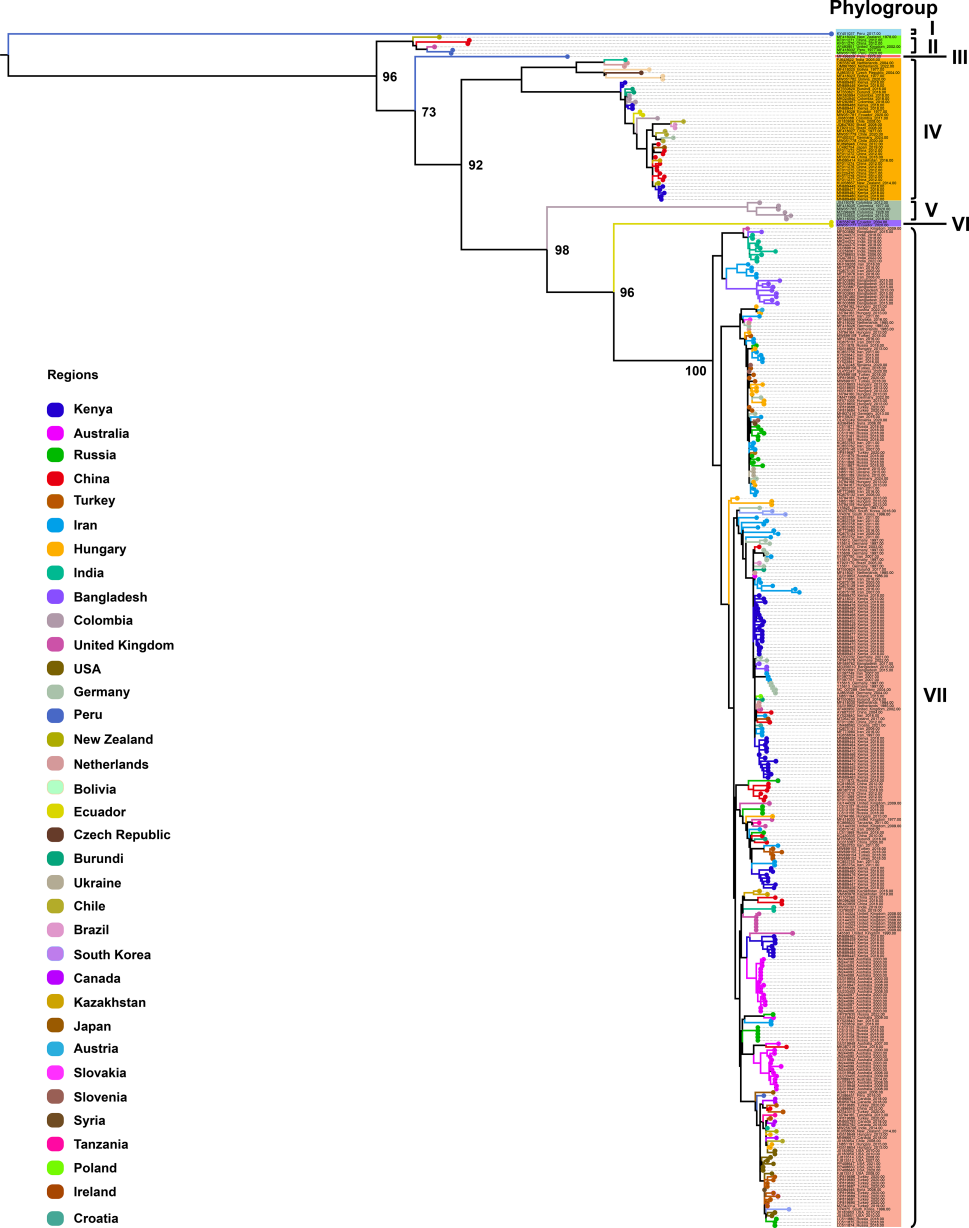
Maximum likelihood (ML) phylogenetic tree inferred from 368 CP gene sequences of PVS. An ML phylogenetic tree was constructed using IQ-TREE v2.3.6 under the TVMe+I+G4 substitution model, with branch support assessed via 1,000 bootstrap replicates. Colors represent the geographic origin of the virus isolates.

To further investigate CP gene diversity on a broader scale, we compiled all available CP sequences (n = 368; length > 800 bp) from 35 countries worldwide and constructed an expanded ML phylogenetic tree (**Figure 2**). This analysis identified seven well-supported phylogroups, designated Phylogroups I–VII. Phylogroup I consisted of a single isolate from Peru, forming an independent lineage. Phylogroup II included six isolates from New Zealand, China, the United Kingdom, and Peru. Phylogroup III comprised a single isolate from Peru. Phylogroup IV represented a geographically diverse clade with 44 isolates originating from Asia (China, India, Kazakhstan, Japan), Africa (Kenya, Burundi), South America (Colombia, Chile, Bolivia, Brazil, Ecuador), Europe (Netherlands, Czech Republic, Germany), and Oceania (New Zealand). Phylogroup V comprised six isolates exclusively from Colombia, forming a distinct, region-specific clade. Phylogroup VI included two isolates from Ecuador, also forming a well-defined, separate lineage. Finally, Phylogroup VII constituted the largest and most geographically diverse clade, encompassing 308 isolates from Africa (Kenya, Burundi, Tanzania), Asia (Iran, Turkey, China, Bangladesh, India, South Korea, Kazakhstan, Syria, Japan), Europe (Russia, Hungary, Germany, United Kingdom, Netherlands, Ukraine, Slovenia, Austria, Croatia, Ireland, Poland, Slovakia), Oceania (Australia, New Zealand), North America (United States, Canada), and South America (Brazil, Chile, Peru). These data further demonstrate the extensive global diversity of PVS and identify Phylogroup VII as the most widespread strain currently circulating worldwide.

### 3.2 Recombination analysis

Recombination analysis of the complete genome sequences of PVS was conducted using the RDP4 software. The results revealed six putative recombinant isolates, with recombination signals supported by several independent detection algorithms implemented in RDP4 (**Table 1**). Only one recombinant originated from parental isolates collected within the same country, whereas the others involved parental sequences from different geographic regions, reflecting frequent interregional genetic exchange (**Table 1**). Similarly, screening of 368 CP gene sequences revealed a single recombinant (India, GU256061), also arising from parental isolates from distinct countries. These findings indicate that most PVS recombination events involve geographically distant isolates, underscoring the role of long-distance virus movement in shaping its genetic diversity.

**Table 1:** Putative recombination events in PVS genomes detected by RDP4.

Event number	Found in	Recombinant	Major parent	Minor parent	Detection methods						
					R	G	B	M	C	S	T
1	1	AJ863510_Czech_Repubic_2004.00	MK024940_Colombia_2018.00	MF418030_Netherlands_1984.00	+	+	+	+	+	+	+
2	3	LN851189_Ukraine_2015.00	PP856220_Gemany_2024.00	MF418031_Kenya_2013.00	+	–	+	+	+	+	+
3	1	MN689463_Kenya_2018.00	MN689482_Kenya_2018.00	MF418031_Kenya_2013.00	–	–	–	–	–	+	+
4	1	LN851194_Poland_2015.00	MF418031_Kenya_2013.00	LN851192_Ukraine_2015.00	+	+	+	+	+	+	+
5	2	MK096268_China_2018.00	MT107560_China_2019.00	FJ813513_USA_2008.00	+	+	+	+	+	+	+
6	2	MK423959_China_2018.00	MT107560_China_2019.00	KU058656_New_Zealand_2014.00	–	–	–	+	–		+

### 3.3 Bayesian evolutionary analysis

**Figure 3:**
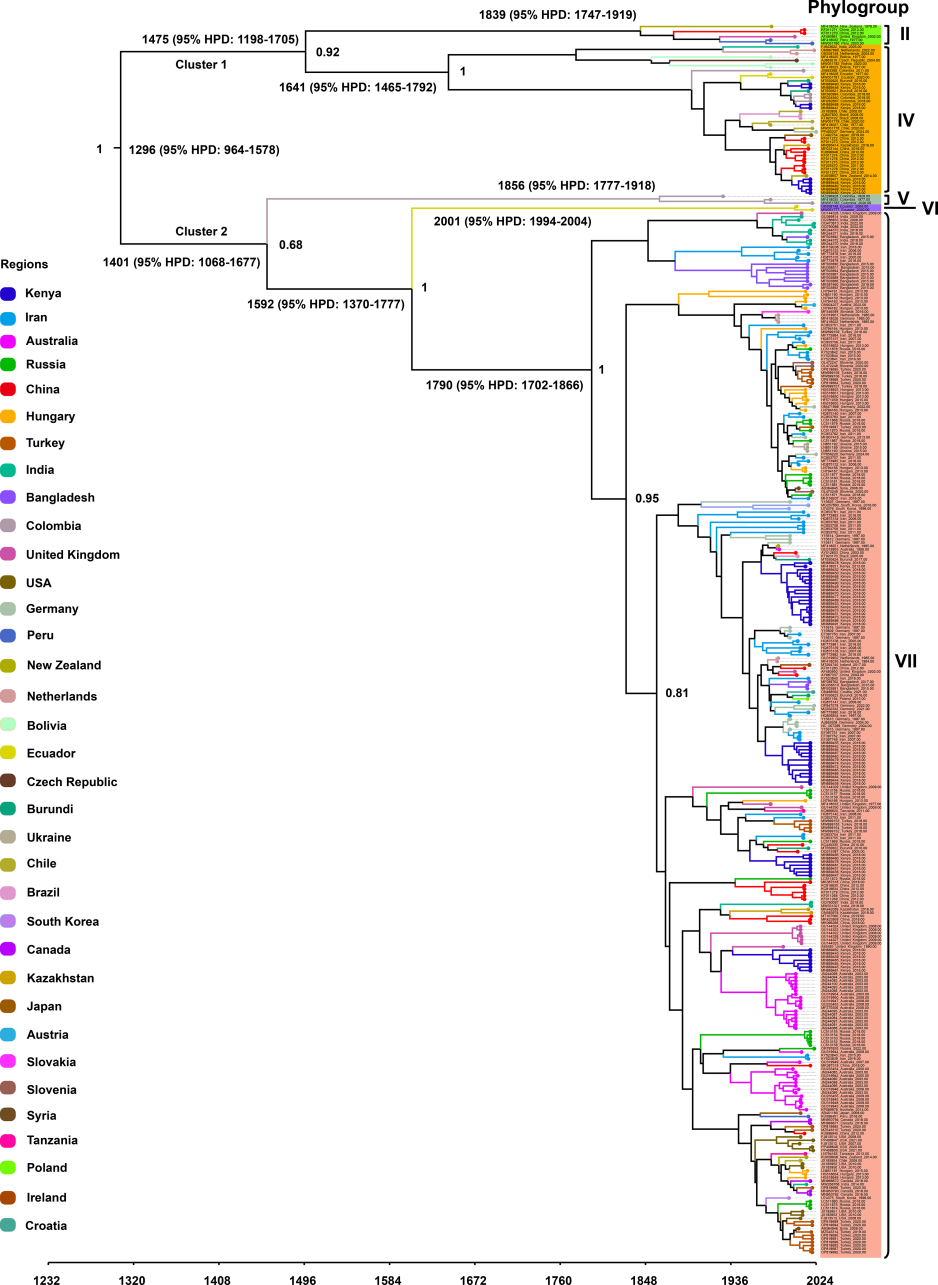
Time-scaled Bayesian phylogenetic tree inferred from 362 PVS CP gene sequences. Colors indicate the countries of virus isolate collection. The estimated times to the most recent common ancestor (tMRCA) for major clades and the posterior probabilities for key nodes are annotated on the tree.

To investigate the temporal dynamics and evolutionary rate of PVS, we removed one confirmed recombinant isolate (GU256061) and five outlier sequences, and subsequently reconstructed a time-scaled Bayesian phylogenetic tree based on the CP gene. As shown in **Figure 3**, the MCC tree was largely congruent with the phylogroup structure inferred from the ML tree, resolving two major clusters: Cluster 1, comprising Phylogroups II and IV, and Cluster 2, consisting of Phylogroups V, VI, and VII. The mean evolutionary rate of the PVS CP gene was estimated at 3.11 × 10^-4^ s/s/y (95% HPD: 2.19 × 10^-4^–4.07 × 10^-4^). The tMRCA of the entire dataset was estimated to be around the year 1296 (95% HPD: 964–1578) (**Figure 3**). The tMRCA for Cluster 1 and Cluster 2 was estimated to be approximately 1475 (95% HPD: 1198–1705) and 1401 (95% HPD: 1068–1677), respectively. Furthermore, the estimated tMRCAs for individual phylogroups were as follows: Phylogroup II: 1839 (95% HPD: 1747–1919), Phylogroup IV: 1641 (95% HPD: 1465–1792), Phylogroup V: 1856 (95% HPD: 1777–1918), Phylogroup VI: 2001 (95% HPD: 1994–2004), and Phylogroup VII: 1790 (95% HPD: 1702–1866) (**Figure 3**). Subsequently, the country-specific tMRCA estimates revealed distinct temporal patterns in the evolutionary history of PVS. A substantial number of countries, New Zealand and China (1308), Burundi, Chile, Ecuador, Germany, India, Kenya, the Netherlands (1310), Peru and the United Kingdom (1311), and Colombia (1313) (**Table 2**). These findings suggest that the 14th century represents a critical period in the early evolutionary history of PVS. In contrast, Bangladesh and Iran (1791), Hungary, Russia, and Turkey (1826), and Australia and South Korea (1860) showed more recent tMRCAs (**Table 2**). The most recent divergence times were estimated for Canada and the USA (1962), Serbia and Slovenia (1978), and Ukraine (2011) (**Table 2**). These results indicate that PVS likely originated in the early 14th century, with subsequent diversification across multiple continents and more recent regional emergence driven by its global spread.

**Table 2:** Inferred time to the most recent common ancestor (tMRCA) of PVS in major countries.

Country	Mean tMRCA	95%HPD	ESS
New Zealand	1308	978-1582	271
China	1308	978-1582	271
Burundi	1310	980-1584	269
Chile	1310	980-1584	269
Ecuador	1310	979-1584	269
Germany	1310	980-1584	269
India	1310	980-1584	269
Kenya	1310	980-1584	269
Netherlands	1310	980-1584	269
Peru	1311	980-1584	272
United Kingdom	1311	980-1584	272
Colombia	1313	977-1591	291
Bangladesh	1791	1702-1866	309
Iran	1791	1702-1866	307
Hungary	1826	1753-1891	318
Russia	1826	1754-1891	315
Turkey	1826	1754-1891	318
Australia	1860	1806-1908	241
South Korea	1860	1804-1909	251
Serbia	1978	1961-1992	424
Slovenia	1978	1961-1992	424
Canada	1962	1936-1985	448
USA	1962	1938-1982	372
Ukraine	2011	2006-2014	5047

### 3.4 Global spatiotemporal dynamics

**Figure 4:**
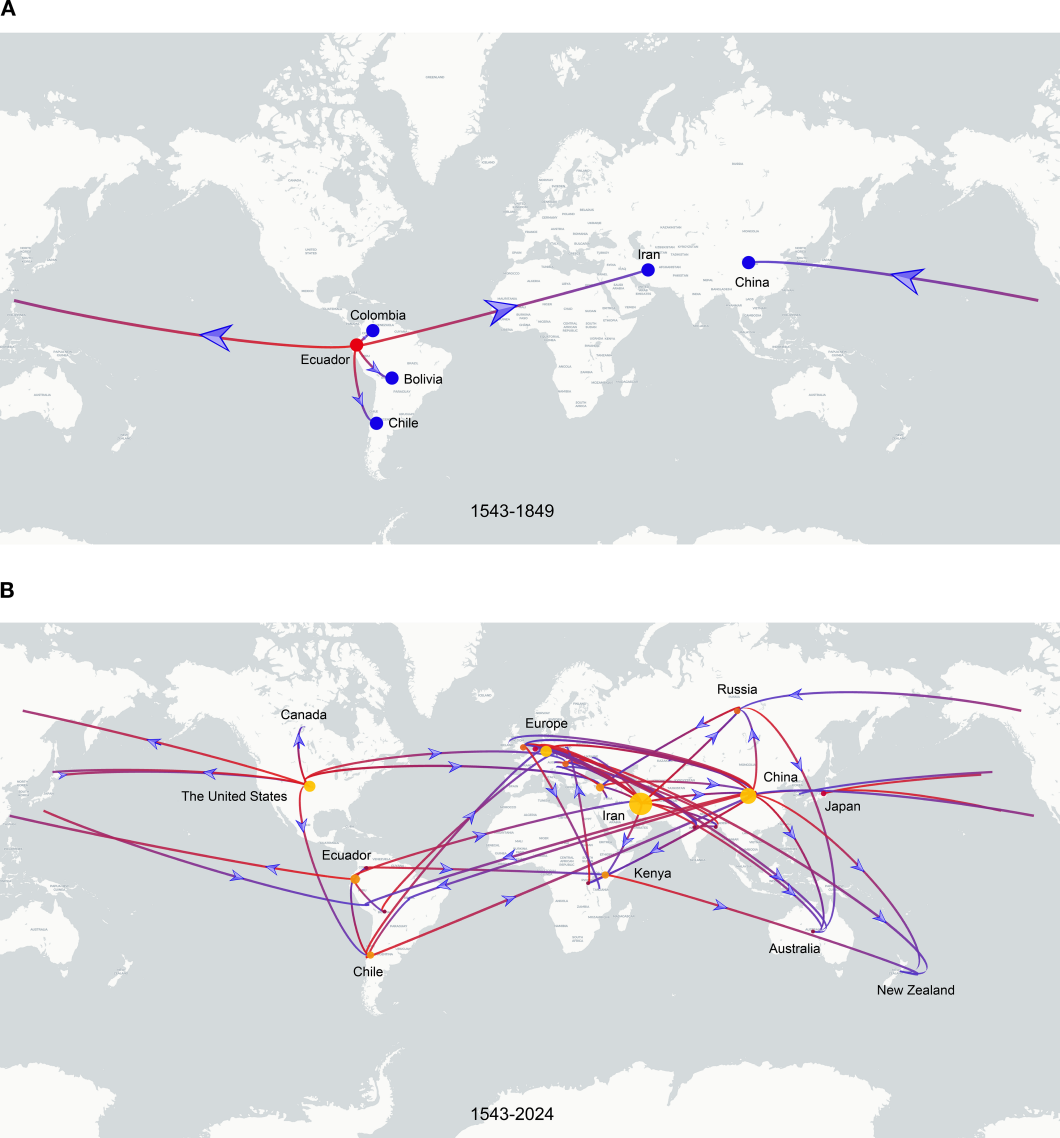
Discrete phylogeographic reconstruction of the global spread of PVS. **(A)** Estimated dissemination routes of PVS between 1543 and 1849. Branches are colored from red (start) to blue (end), with red and blue dots marking the inferred source and target countries, respectively. Blue arrows indicate the direction of viral spread. **(B)** Global dissemination of PVS between 1543 and 2024. The start and end of the branches are coloured in red and blue, respectively. The size of the yellow clusters represents the cumulative lineage counts. The global transmission routes of PVS are displayed by filtering for Bayes factors >5.

**Figure 5:**
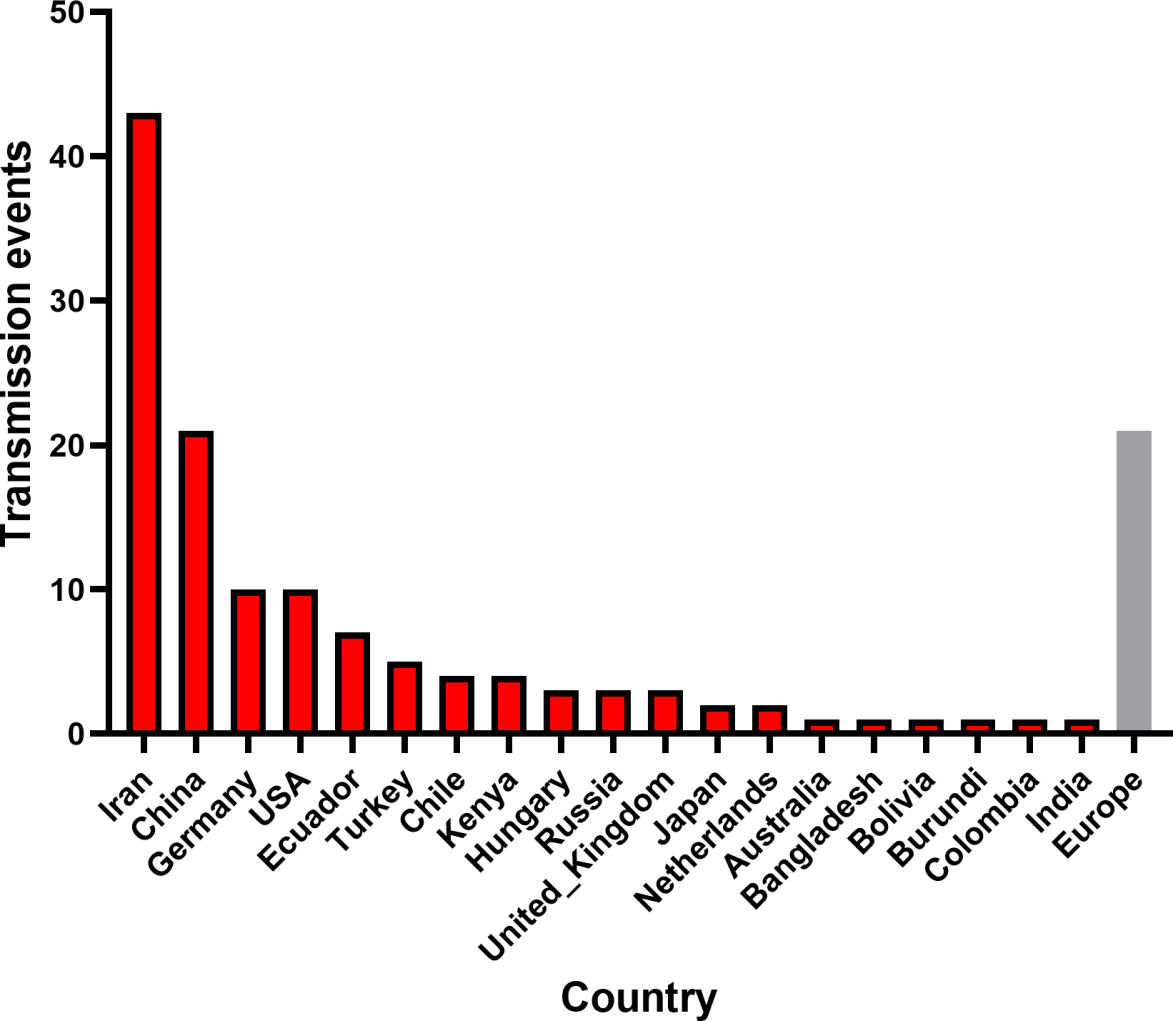
Global transmission events of PVS. The number of cross-border PVS transmission events with Bayes factor support >5 was quantified for each source country. The gray bar represents the combined dissemination events originating from European countries, including Germany, the United Kingdom, the Netherlands, Russia, and Hungary.

**Figure 6:**
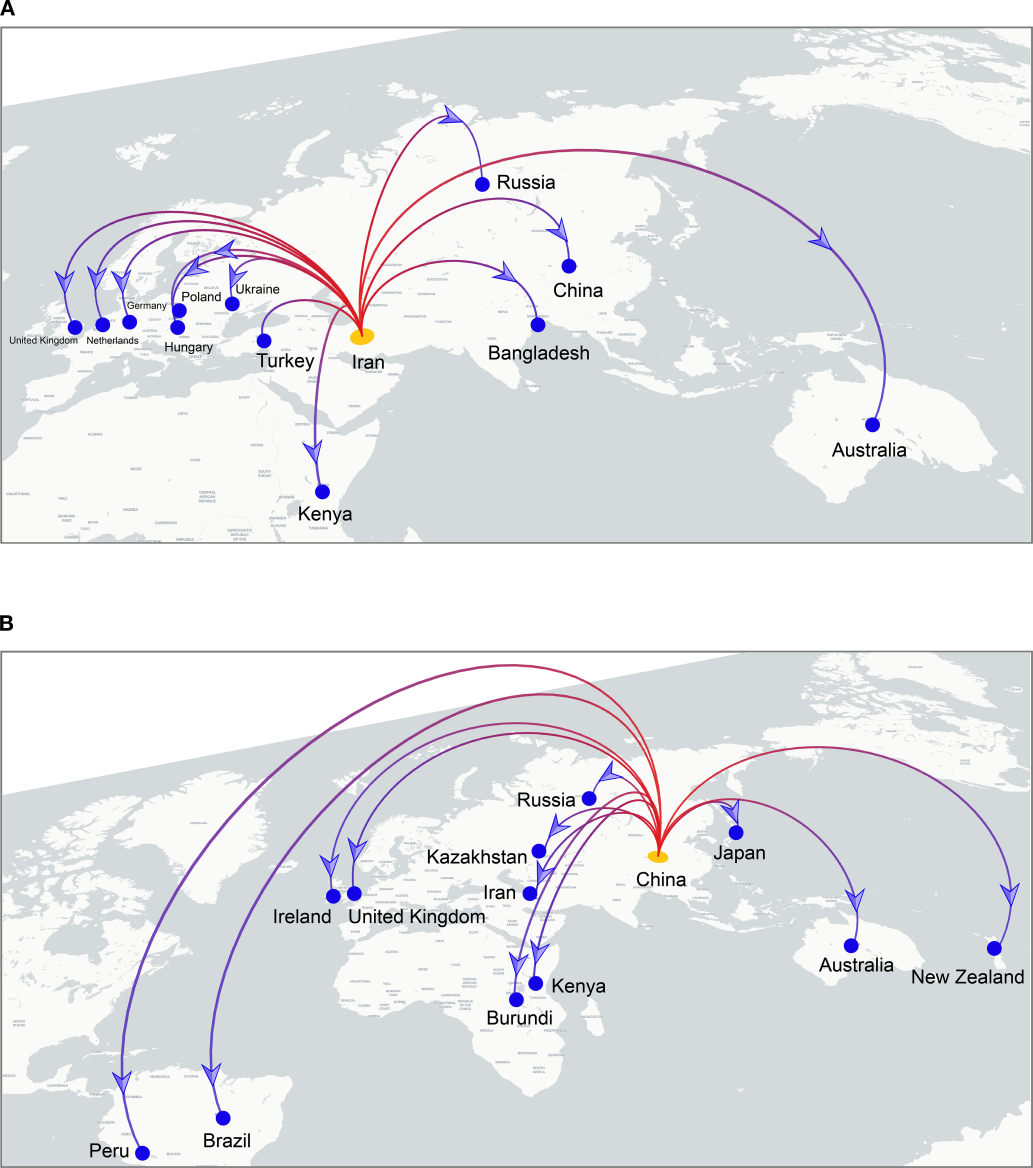
Cross-border dissemination routes originating from Iran **(A)** and China **(B)**. Branches are colored from red (start) to blue (end), with yellow and blue dots marking the inferred source and target countries, respectively. Blue arrows indicate the direction of viral spread.

To investigate the global spatiotemporal dynamics of PVS, we reconstructed its spatial transmission routes using a Bayesian phylogeographic framework based on the CP gene dataset (n = 362) and visualized the results using the Spread.gl software. The time-scaled phylogeographic reconstruction indicated that Ecuador is likely the center of origin for PVS around 1543 (**Figure 4A**; **Supplementary Table S3**). The earliest statistically supported dissemination event occurred in the 16th century, with viral dispersal from Ecuador to China in 1543. During the 17th to 18th centuries, the virus continued to spread from Ecuador to neighboring South American countries including Colombia, Bolivia, and Chile, as well as intercontinentally to Iran in Asia (**Figure 4A**; **Supplementary Table S3**). In the 19th century, secondary spread from China, Iran, and Chile facilitated further introductions into geographically distant regions such as New Zealand, the United Kingdom, and Bangladesh, marking the expansion of PVS into Oceania, Europe, and South Asia. This was followed by a marked acceleration of global spread in the 20th century, with PVS reaching a wide range of countries across all continents (**Figure 4B**; **Supplementary Table S3**). To identify key hubs facilitating PVS dissemination, we quantified the number of supported transmission events (BF > 5) originating from each country. The results revealed that Iran and China served as major secondary epicenters, each acting as significant sources of subsequent viral introductions (**Figure 5**). Iran accounted for the highest number of outward transmission events (n = 43), primarily spreading PVS to Europe, Asia, and Africa during the 19th and 20th centuries. China followed with 21 intercontinental export events, contributing to the spread of PVS across Asia, Africa, Europe, Oceania, and back to South America (**Figures 5**, **6A, B**). In addition, several European countries, including Germany, the United Kingdom, the Netherlands, Russia, and Hungary, emerged as important transmission hubs, collectively contributing 21 export events to countries in Asia, Africa, Europe, and Oceania in the 20th and 21st centuries (**Supplementary Figure S2A**; **Supplementary Table S3**). The United States (n = 10) was also identified as a key dissemination center, facilitating viral spread to neighboring Canada as well as to regions in Europe, Asia, and South America in the 20th and 21st centuries (**Supplementary Figure S2B**; **Supplementary Table S3**).

### 3.5 Genetic diversity of PVS populations across global regions

Analysis of CP gene sequences using DnaSP v.6.12.03 revealed that PVS populations exhibit consistently high haplotype diversity (Hd > 0.97) across all regions, with the highest values in Asia (Hd = 0.9984) and Africa (Hd = 0.997) (**Table 3**). Nucleotide diversity (π) varied more markedly, ranging from 0.01754 in North America to 0.14017 in South America, the latter showing the greatest overall diversity (π = 0.14017; k = 103.166; S = 297) (**Table 3**). Pairwise comparisons of population differentiation further indicated contrasting patterns among regions (**Table 4**). The lowest genetic differentiation was observed between Asia and Europe (Fst = 0.02682; Nm = 9.07), followed by moderate levels between Asia–Africa (Fst = 0.05088; Nm = 4.66) and Europe–Africa (Fst = 0.10860; Nm = 2.05), while comparisons involving Oceania showed intermediate structure. In contrast, all comparisons with the Americas exhibited high to very high differentiation (Fst > 0.25) and limited gene flow (Nm < 1), with the strongest divergence detected between North and South America (Fst = 0.50049; Nm = 0.25) (**Table 4**). Collectively, these results demonstrate that PVS possesses high global genetic diversity with pronounced regional structuring, characterized by close connectivity among Old-World populations and strong isolation of New-World populations.

**Table 3:** Population genetic parameters of PVS isolates from different regions worldwide.

Region	*N*	*H*	*Hd*	*S*	*η*	*K*	*π*
Europe	93	74	0.992	285	394	41.159	0.05749
Asia	132	118	0.9984	346	496	63.909	0.08173
Africa	62	57	0.997	245	299	62.354	0.08035
North America	15	12	0.971	55	58	13.733	0.01754
South America	30	28	0.995	297	442	103.166	0.14017
Oceania	36	30	0.989	231	288	36.297	0.04636

*n*, number of isolates; *h*, number of haplotypes; *Hd*, haplotype diversity; *S*, number of variable sites; *η*, total number of mutations; *k*, average number of nucleotide differences; *π*, nucleotide diversity (Pi).

**Table 4:** Genetic differentiation and gene flow among PVS populations from different global regions.

Region comparison		Fst	Nm
Asia	Europe	0.02682	9.07
	Africa	0.05088	4.66
	North America	0.28125	0.64
	South America	0.30210	0.58
	Oceania	0.13650	1.58
Europe	Africa	0.10860	2.05
	North America	0.36608	0.43
	South America	0.38093	0.41
	Oceania	0.18170	1.13
Africa	North America	0.33510	0.50
	South America	0.28153	0.64
	Oceania	0.17404	1.19
North America	South America	0.50049	0.25
	Oceania	0.42999	0.33
South America	Oceania	0.41506	0.35

Fst (Fixation index) measures the degree of genetic differentiation among populations, with values ranging from 0 (no differentiation) to 1 (complete differentiation), and higher values indicating stronger genetic divergence. Nm (Number of migrants per generation) estimates the level of gene flow, where Nm > 1 suggests sufficient migration to counteract the effects of genetic drift, while Nm < 1 indicates restricted gene flow that may lead to significant population differentiation.

## 4 Discussion

In this study, we provide a comprehensive analysis of the evolutionary dynamics, phylogeography, and global dissemination of PVS based on complete genome and CP gene sequences. The updated Latin numeral-based phylogroup classification refined previous schemes and revealed substantial genetic diversity among PVS isolates. Both geographically restricted lineages, such as those confined to Colombia and Ecuador, and widely distributed strains, including globally pervasive phylogroups, were identified, highlighting the complex evolutionary structure of the virus. These findings emphasize that PVS evolution is shaped by both regional diversification and long-distance dissemination, reflecting the interplay of natural evolutionary processes and human-mediated spread.

The genome-based ML tree, constructed from 152 complete sequences across 29 countries, resolved two major clusters encompassing four phylogroups (I–IV). Phylogroup I consisted exclusively of Colombian isolates, forming a distinct region-specific lineage, while Phylogroup IV emerged as the most prevalent and geographically widespread group, including multiple country-specific monophyletic clades suggestive of localized diversification. In contrast, the CP gene phylogeny based on the same 152 genomes revealed topological discrepancies and classified isolates into five phylogroups (I–V), indicating that single-gene analyses may not fully capture the evolutionary patterns inferred from complete genomes due to gene-specific evolutionary constraints. To enhance phylogenetic resolution and assess broader global diversity, we analyzed an expanded CP dataset of 368 sequences from 35 countries, which identified seven well-supported phylogroups (I–VII). Among these, Phylogroups V and VI were restricted to Colombia and Ecuador, respectively, representing geographically limited lineages, whereas Phylogroup VII encompassed the majority of isolates and exhibited the widest global distribution. Integrating results from both genome and CP gene datasets, our analyses indicate that PVS currently occurs in 35 countries across six continents, demonstrating a complex pattern of both localized diversification and widespread dissemination. These results highlight the global spread and complex evolutionary structure of PVS, emphasizing the coexistence of geographically restricted lineages and widely disseminated strains.

Recombination is a key evolutionary mechanism that enhances viral genetic diversity and adaptability (Pérez-Losada et al., 2015). By enabling the exchange of genetic material during mixed infections, it can produce novel variants with improved fitness, expanded host range, or increased resistance to host defenses (Sanjuan and Domingo-Calap, 2021; Retel et al., 2019). Recombination is a well-documented mechanism in plant viruses, facilitating rapid adaptation to changing environments and agricultural practices (Nagy, 2008; Krupovic et al., 2015). In our analysis of 152 complete PVS genomes, six recombinant isolates were identified, most involving parental sequences from geographically distinct regions, highlighting the role of interregional virus movement, likely mediated by international trade of infected plant material. Screening of 368 CP gene sequences revealed only a single recombinant, indicating that recombination is relatively rare but potentially significant for viral diversification. Comparisons with previous studies show discrepancies in detected recombinants, reflecting differences in dataset size, sequence diversity, algorithm sensitivity, and software versions (Santillan et al., 2018; Topkaya et al., 2023). These findings underscore the importance of dataset scope and methodological considerations in recombination detection and emphasize that recombination, even if infrequent, contributes to the genetic diversity and evolutionary potential of PVS.

In the present study, the Bayesian time-scaled phylogenetic analysis of the PVS CP gene, conducted after the exclusion of one recombinant and several outlier sequences, yielded an estimated evolutionary rate of approximately 3.11 × 10^-4^ s/s/y (95% HPD: 2.19 × 10^-4^–4.07 × 10^-4^). This estimate is consistent with previous studies based on the CP gene, which reported similar substitution rates of 3.32 × 10^-4^ s/s/y (95% HPD: 1.33 × 10^-4^–5.58 × 10^-4^) and 2.71 × 10^-4^ s/s/y (95% HPD: 1.37 × 10^-4^–4.10 × 10^-4^) (Duan et al., 2018; Santillan et al., 2018). The estimated time to tMRCA of the analyzed PVS CP sequences was dated to approximately 1296 (95% HPD: 964–1578). This estimate closely matches previous results, which inferred tMRCAs of 1325 (95% HPD: 762–1743) and 1067 CE 95% HPD: 68–1369) (Duan et al., 2018; Santillan et al., 2018). Divergence times for the two major phylogenetic clusters were estimated at 1475 (Cluster 1; 95% HPD: 1198–1705) and 1401 (Cluster 2; 95% HPD: 1068–1677), while individual phylogroups showed staggered emergence between the 17th and 21st centuries. The country-specific tMRCA estimates revealed distinct temporal patterns that shed light on the evolutionary history and possible origin of PVS. Among the earliest divergence times were those for New Zealand and China (1308), Colombia (1313), Peru and the United Kingdom (1311), and the Netherlands (1310). The fact that New Zealand and China displayed the earliest lower HPD bounds suggests that they may harbor ancient viral lineages, potentially reflecting early introductions or persistence of ancestral PVS variants. However, given that Peru and Colombia are part of the Andean region, the center of origin and domestication for cultivated potato, and considering the overall pattern of phylogenetic structure and historical crop dissemination, South America remains the most plausible center of origin for PVS. The early appearance of PVS in distant regions such as Europe, Asia, and Oceania implies rapid dissemination following the global movement of potato, particularly through the Columbian Exchange (Nunn and Qian, 2010). In contrast, more recent divergence times observed in countries such as Bangladesh and Iran (1791), Hungary, Russia, and Turkey (1826), and Australia and South Korea (1860) indicate later introductions likely linked to colonial-era agricultural expansion. The most recent tMRCA estimates for Canada and the USA (1962), Serbia and Slovenia (1978), and Ukraine (2011) likely reflect contemporary virus movement through international seed trade. These findings indicate that PVS has undergone long-term diversification closely associated with the global spread of cultivated potato, shaped by both early dissemination events and more recent introductions linked to historical and contemporary agricultural practices.

The cultivated potato originated in the Andean region of South America, particularly in present-day southern Peru and northwestern Bolivia, where it was domesticated approximately 7,000 years ago. Following its domestication, the potato underwent gradual dissemination, especially after the Columbian Exchange in the 16th century, which introduced the crop to Europe, Asia, and other continents. This global expansion of the host plant likely facilitated the parallel spread and diversification of associated viruses, including PVS (Spooner et al., 2005; Nunn and Qian, 2010; Jones, 2009). Although PVS was first reported infecting potato crops in Europe in 1952 (Santillan et al., 2018), our Bayesian phylogeographic reconstruction based on the CP gene dataset reveals that Ecuador, within the Andean region, is the most likely center of origin for PVS around 1543. This finding provides robust statistical support for the hypothesis that PVS originated in the Andean highlands, the same center as its primary host, thereby reinforcing the concept of virus-host co-evolution and co-dispersal. The temporal reconstruction further indicates that the earliest supported intercontinental transmission of PVS occurred in the 16th century, from Ecuador to China, followed by both regional spread to neighboring South American countries and long-distance dissemination to Iran in the 17th–18th centuries. A marked acceleration of global spread occurred during the 19th and 20th centuries, coinciding with increased international crop exchange and agricultural trade. These results highlight the critical role of human-mediated movement of plant materials in facilitating the historical and contemporary spread of PVS, supporting the broader paradigm of human-driven plant virus globalization. Moreover, our findings identify Iran and China as major secondary hubs of global transmission, particularly during the 19th and 20th centuries. This contrasts with previous conclusions by Duan et al., who proposed that Europe acted as the primary dissemination hub of PVS in the 19th century (Duan et al., 2018). To clarify this discrepancy, we analyzed intercontinental transmission events originating from key European countries (Germany, the United Kingdom, the Netherlands, Russia, and Hungary) and found that although Europe also served as an important hub, its role in mediating viral dissemination was primarily concentrated in the 20th and 21st centuries rather than the 19th. Additionally, our analysis reveals that the United States has also emerged as a significant transmission hub, contributing to the globalization of PVS through its dissemination to neighboring and distant regions. Collectively, these findings underscore the temporally dynamic and geographically shifting nature of viral dissemination networks shaped by historical and modern agricultural practices.

To complement phylogenetic and phylogeographic inferences, we further examined the population genetic diversity and structure of PVS across six continents (Europe, Asia, Africa, North America, South America, and Oceania) using CP gene sequences. Estimates of molecular diversity indices (h, Hd, S, η, k, and π) revealed that South America harbors the highest haplotype and nucleotide diversity, consistent with its status as the center of origin and long-term diversification of PVS. In contrast, Africa and Oceania exhibited the lowest levels of diversity, likely reflecting more recent and limited introductions via international seed trade. Genetic differentiation and gene flow analyses provided further resolution: high Fst values between South America and other regions indicated strong genetic subdivision and restricted connectivity, whereas lower Fst and higher Nm between Asia and Europe suggested relatively frequent exchanges, consistent with historical and modern agricultural trade routes. Together, these results demonstrate that while PVS evolution is deeply rooted in the Andean region, its contemporary genetic structure reflects both ancient diversification and recent intercontinental movement.

In conclusion, this study provides a comprehensive analysis of the evolutionary and spatiotemporal dynamics of PVS based on complete genome and CP gene sequences. Phylogenetic reconstruction refined the phylogroup classification and revealed substantial genetic diversity shaped by regional evolutionary patterns. Phylogeographic analyses suggested that Ecuador is the likely center of origin, with intercontinental spread beginning in the 16th century and accelerating during the 19th and 20th centuries. Iran and China emerged as major secondary hubs facilitating global dissemination. Population genetic analyses further showed that South America harbors the highest diversity, supporting its role as the center of origin, while reduced variation in Africa and Oceania reflects more recent introductions. These findings enhance our understanding of PVS evolution, population structure, and global spread, and offer valuable insights for its worldwide monitoring and management.

## Data Availability

All sequence data analyzed in this study were obtained from the GenBank database, with accession numbers listed in the **Supplementary Materials**.
