# Supplementary figures and images for: Origin, evolution, dispersal and global population genetic structure of Carlavirus sigmasolani
Source: Front Plant Sci. 2025 Sep 24;16:1667771. doi: 10.3389/fpls.2025.1667771 (PMC12504366; doi:10.3389/fpls.2025.1667771)

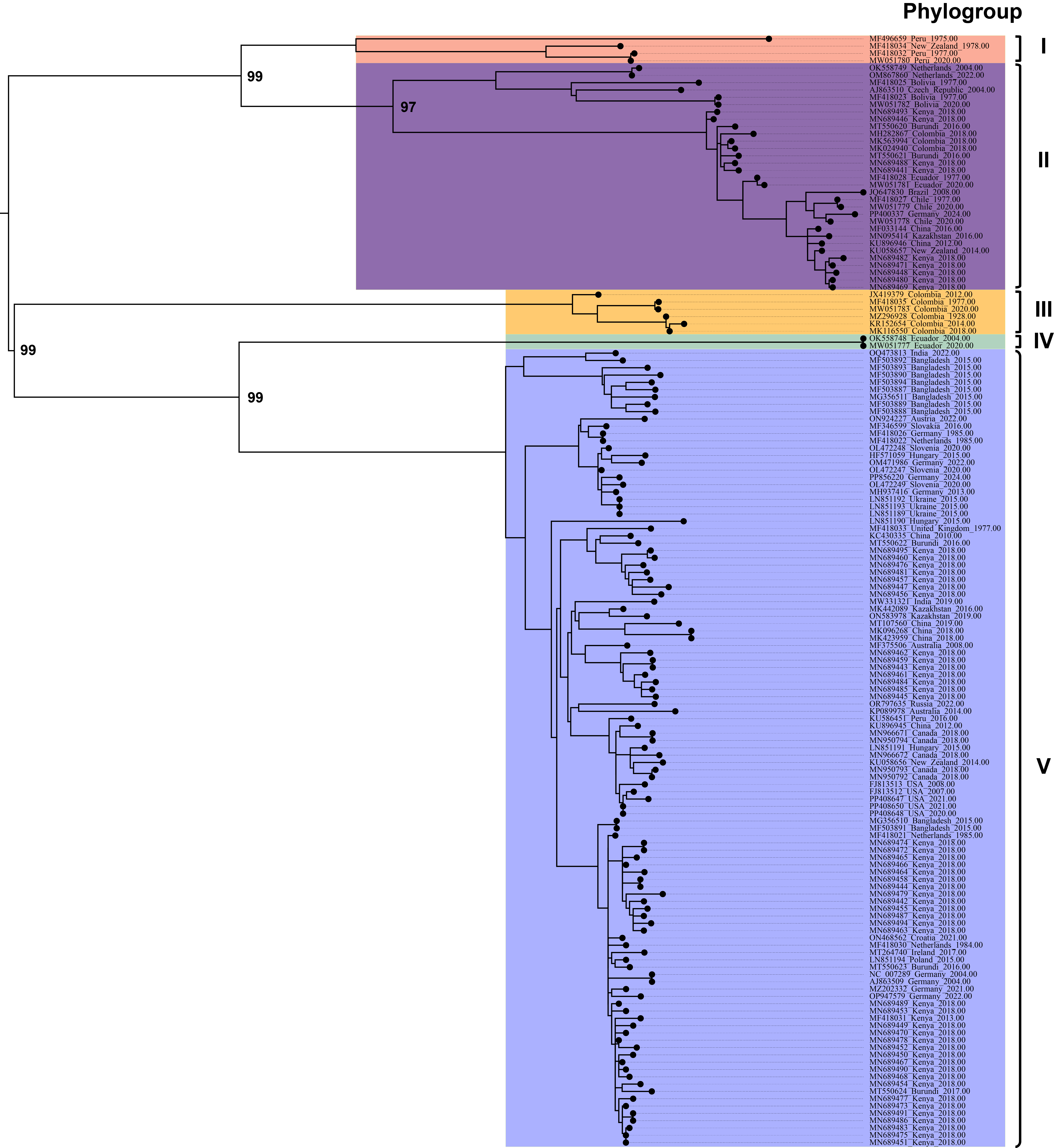

Supplement: Supplementary file 4 [file Image1.tif]
